# Supplementary figures and images for: GABA and Combined GABA with GAD65-Alum Treatment Alters Th1 Cytokine Responses of PBMCs from Children with Recent-Onset Type 1 Diabetes
Source: Biomedicines. 2023 Jul 10;11(7):1948. doi: 10.3390/biomedicines11071948 (PMC10377053; doi:10.3390/biomedicines11071948)

# Supplemental Figure S1

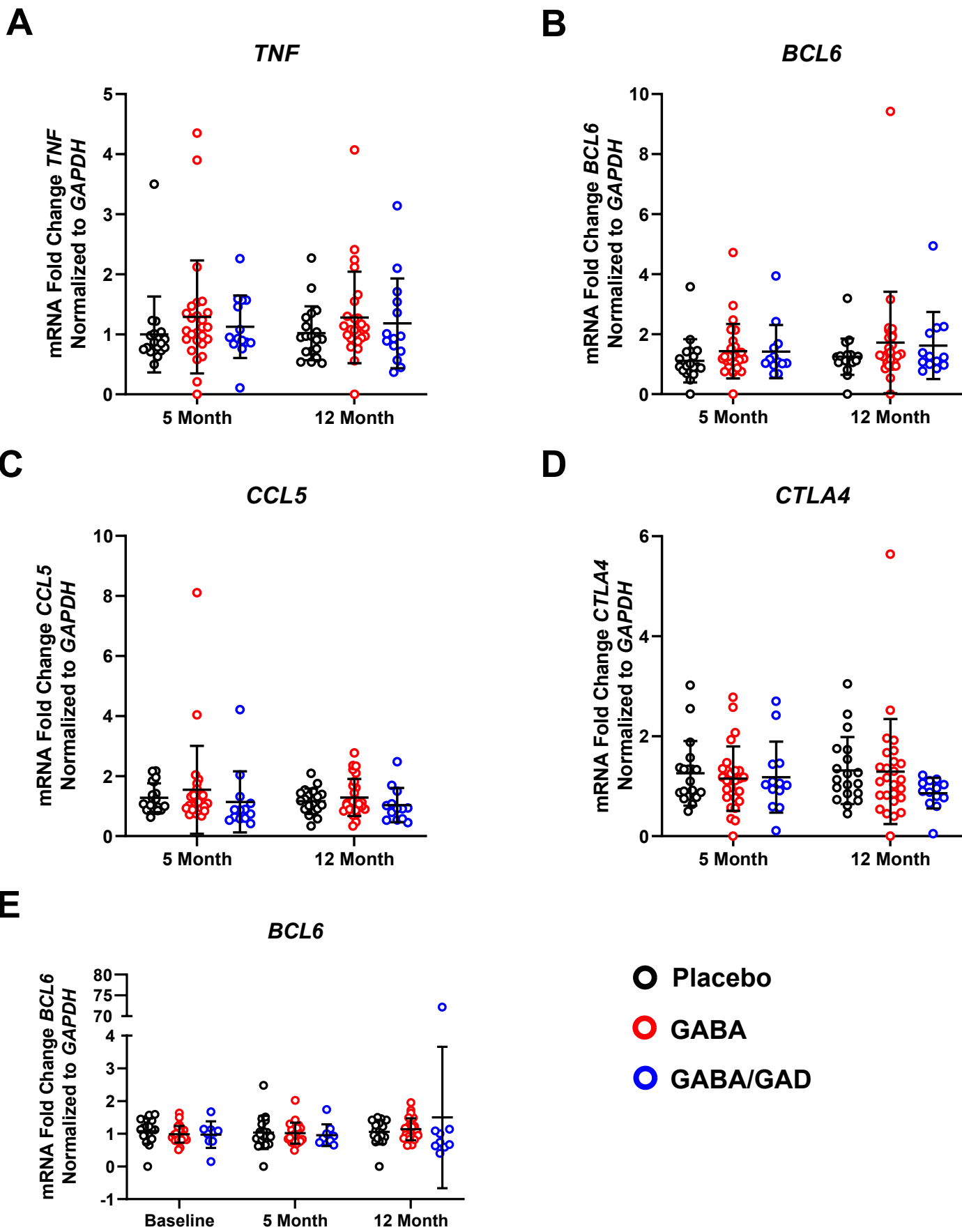

# Supplemental Figure S2

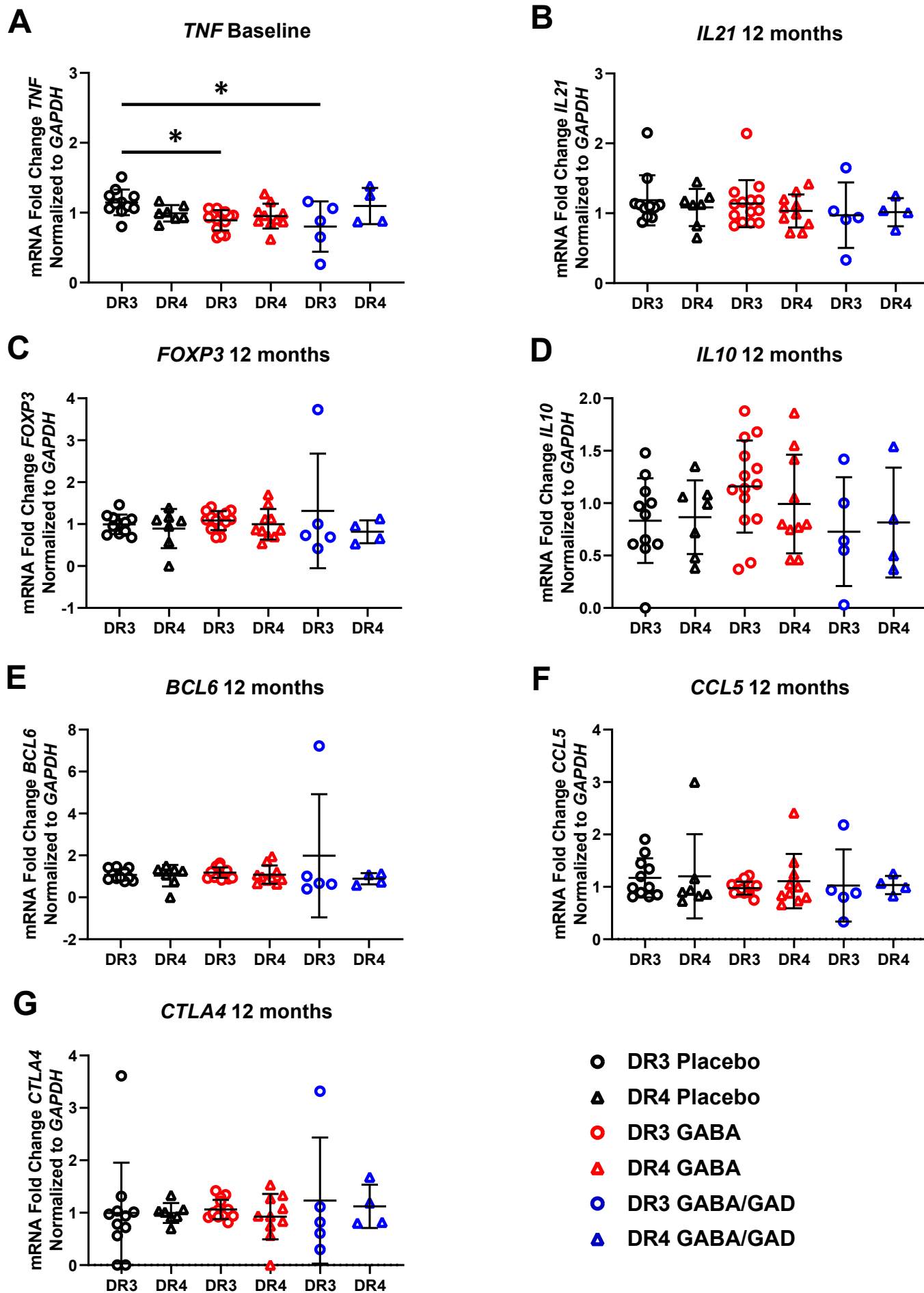

# Supplemental Figure S3

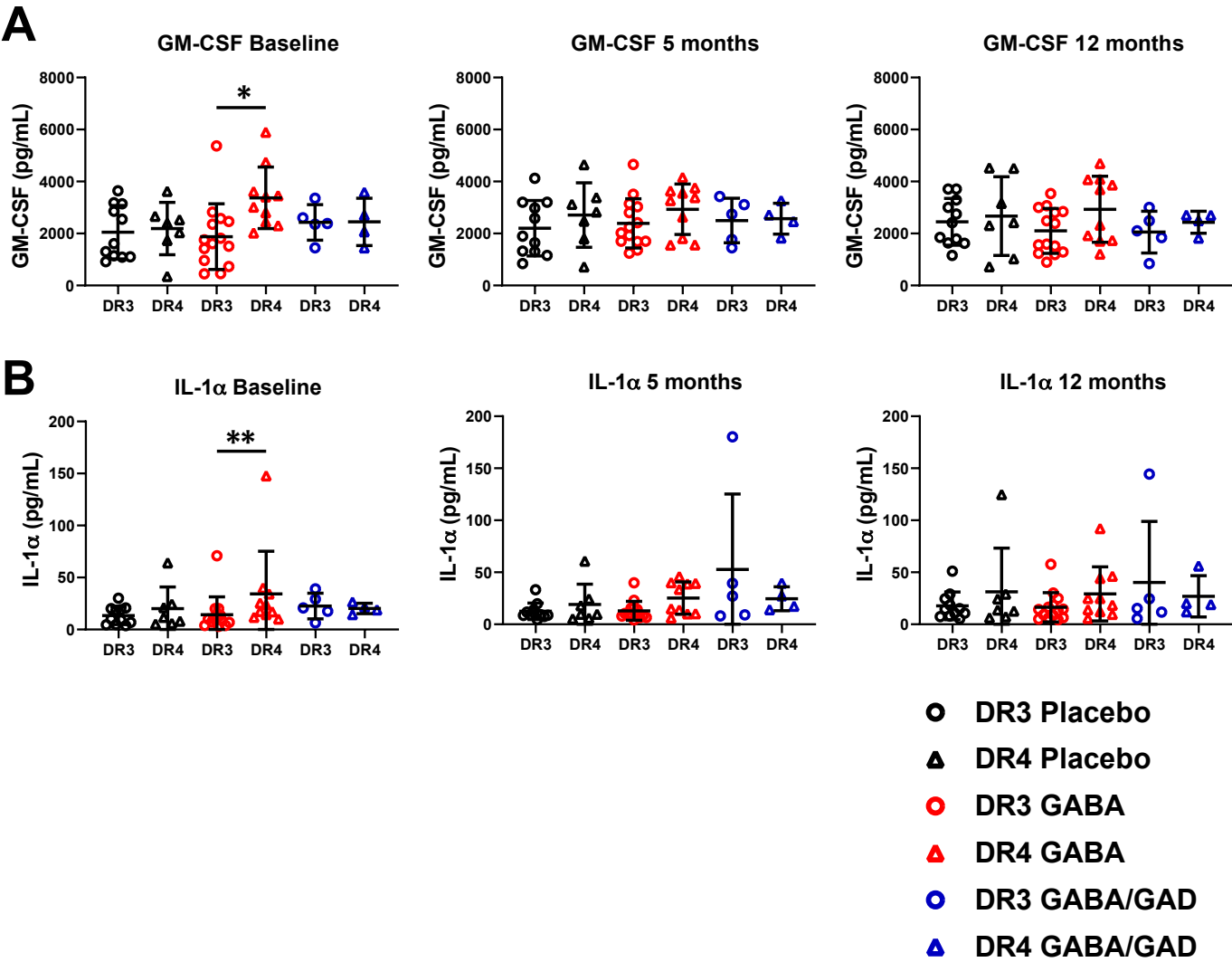

Supplement: Supplementary file 1 [file biomedicines-11-01948-s001.zip › biomedicines-2434232-supplementary.pdf]
